# Lateral frontal cortex volume reduction in Tourette syndrome revealed by VBM

**DOI:** 10.1186/1471-2202-13-17

**Published:** 2012-02-14

**Authors:** Matthias Wittfoth, Sarah Bornmann, Thomas Peschel, Julian Grosskreutz, Alexander Glahn, Nadine Buddensiek, Hartmut Becker, Reinhard Dengler, Kirsten R Müller-Vahl

**Affiliations:** 1 Department of Neurology, Hannover Medical School, Hannover, Germany; 2 NeuroImaging and Clinical Applications (NICA), Hannover, Germany; 3 Clinic of Psychiatry, Socialpsychiatry and Psychotherapy, Hannover Medical School, Hannover, Germany; 4 Department of Neurology, University of Jena, Jena, Germany; 5 Institute of Diagnostic and Interventional Neuroradiology, Hannover Medical School, Hannover, Germany

**Keywords:** Gilles de la Tourette syndrome, Voxel-based morphometry, Inferior frontal gyrus, Amygdala, Obsessive-compulsive disorder, Attention deficit hyperactivity disorder

## Abstract

**Background:**

Structural changes have been found predominantly in the frontal cortex and in the striatum in children and adolescents with Gilles de la Tourette syndrome (GTS). The influence of comorbid symptomatology is unclear. Here we sought to address the question of gray matter abnormalities in GTS patients *with *co-morbid obsessive-compulsive disorder (OCD) and/or attention deficit hyperactivity disorder (ADHD) using voxel-based morphometry (VBM) in twenty-nine adult actually unmedicated GTS patients and twenty-five healthy control subjects.

**Results:**

In GTS we detected a cluster of decreased gray matter volume in the left inferior frontal gyrus (IFG), but no regions demonstrating volume increases. By comparing subgroups of GTS with comorbid ADHD to the subgroup with comorbid OCD, we found a left-sided amygdalar volume increase.

**Conclusions:**

From our results it is suggested that the left IFG may constitute a common underlying structural correlate of GTS with co-morbid OCD/ADHD. A volume reduction in this brain region that has been previously identified as a key region in OCD and was associated with the active inhibition of attentional processes may reflect the failure to control behavior. Amygdala volume increase is discussed on the background of a linkage of this structure with ADHD symptomatology. Correlations with clinical data revealed gray matter volume changes in specific brain areas that have been described in these conditions each.

## Background

Gilles de la Tourette syndrome (GTS) is a complex chronic motor and vocal tic disorder with childhood onset. However, the majority of patients, in addition, suffer from different comorbid disorders, most often obsessive-compulsive disorder (OCD) and attention deficit hyperactivity disorder (ADHD) [1,2]. On average, 20-60% of all GTS patients have comorbid OCD, while about 50% suffer from ADHD [3]. In contrast, GTS "only" - without any comorbidity - occurs in only 10 to 15% [4,5]. Previous structural imaging studies investigating gray and white matter abnormalities in GTS revealed conflicting results. It has been suggested that these inconsistent data are mainly related to differences in patients' age, gender, handedness, medication status, comorbidities, imaging techniques, and analysis strategy. Despite these discrepancies, there is substantial evidence that structural alterations in several brain regions are indeed related to the pathology of GTS (for an overview, see also [6]). Most of these changes corroborate the hypothesis of alterations in cortico-striato-thalamo-cortical circuits [7-10]. In particular, there is evidence for reduced basal ganglia volumes [10], a volume decrease of the amygdala in adults [11,12], but increased volumes in children [12], and an increase of gray matter volumes in the mesencephalon [13,14] and in dorsolateral prefrontal regions in children, but not in adults [9]. In a treatment-naïve boys-only group without comorbidities, increased volumes of the putamen bilaterally and the corpus callosum (subregion 3) were found [15]. A study using diffusion tensor imaging (DTI), a method that allows conclusions about the microstructural organization based on water diffusion properties [16], provided additional evidence for white matter abnormalities of somatosensory pathways in GTS adults [17]. By means of high-resolution structural MRI, recent studies showed cortical thinning in large areas of the frontal and parietal lobe in children with GTS [18], and reduced cortical thickness in motor, premotor, prefrontal and lateral orbitofrontal areas in adult patients suffering from GTS [19]. Available morphometric studies strongly suggest that structural patterns in children are quite opposite to those in adults indicating the occurrence of neuroplastic developmental processes during the course of the disease. In line with this assumption are findings of increased prefrontal volumes with associated decreased size of the corpus callosum in GTS children [20,21] - which are thought to reflect compensatory mechanisms in order to facilitate suppression of tics [9,22] - but decreased dorsal prefrontal and increased corpus callosum volumes in GTS adults which are possibly associated with symptom persistence into adulthood [9,21,23]. In a recent study from our group, we used voxel-based morphometry (VBM) and magnetization transfer imaging (MTI) to investigate actually unmedicated adult GTS "only" patients and found structural differences predominantly in prefrontal areas, stressing a more fronto-striatal dysfunction rather than a distinct basal ganglia involvement [24].

VBM has been proven to be a powerful method for the in vivo study of human brain structures [25,26]. This technique relies on the segmentation of magnetic resonance (MR) images into different tissue types (e.g. gray matter, white matter, and cerebrospinal fluid) using probability measures based on image intensities [27]. It is a semi-automated, unbiased technique that is based on a voxel-wise analysis without the need for a priori hypotheses. Here we sought to address the question of gray matter abnormalities in adult GTS patients *with *comorbid OCD and/or ADHD compared to healthy controls using VBM. More specifically, we applied a sophisticated computational image analysis approach both to compare regional volumes of gray matter throughout the brain [28] and to test whether there is a common pattern of structural brain alteration occurring in patients with GTS plus OCD as well as in those with comorbid ADHD.

## Results

### Clinical assessments

There were no differences in sex, age, and handedness between GTS patients and normal controls. Comparing clinical parameter, the IQ (MWT-B) was found significantly lower in GTS patients compared to healthy controls (t = 2.3; df = 51; *p *= 0.03). However, none of the patients had an IQ below 92. Results of clinical assessments regarding severity of tics, OCD, ADHD, depression, and anxiety differed significantly between GTS patients and normal controls (see Table 1).

**Table 1:** Demographical and clinical characteristics

	GTS plus (n = 8)	GTS- OCD (n = 17)	GTS-ADHD (n = 4)	Healthy control group (n = 24)	p
**Age**	Mean 30.7 (9), range 18-49			Mean 30.6 (10.9), range 18-59	.97

**Sex**		male		male	-

**Handedness**		right		right	-

**IQ**		105.6 (13.3)		114 (15)	.03

**RVTRS**		8 (4.6)		0	-

**YGTSS**		35.7 (17.6)		0	-

**Y-BOCS obsessions**	6.5 (5.5)	10.5 (6.2)	0	0	-

**Y-BOCS compulsions**	9.6 (5.4)	10.4 (4.4)	0	0	-

**WURS-K**	38 (9.2)	19.9 (9)	29.3 (10.2)	13.5 (9.7)	.001

**CAARS**	5.6 (2.4)	4.9 (1.6)	4.3 (1.1)	4 (1.6)	.04

**ADHD symptom list part 1**	7.2 (1.5)	2.6 (2.1)	6.3 (2)	1 (1.8)	.003

**ADHD symptom list part 2**	6.2 (2.4)	0.7 (1.1)	5 (2.6)	1 (1.3)	.001

**BDI**	13.2 (9.1)	4.2 (3.6)	1 (1.7)	1.9 (2.4)	.001

**STAI- 1**	57.6 (7.8)	39.8 (10.5)	27.3 (1.5)	29.5 (6.3)	.001

**STAI- 2**	55 (10.2)	43.2 (12.7)	25.7 (5.5)	31 (8)	.003

All additional values in parenthesis are standard deviations.

### Differences in GM volumes between patients with GTS and healthy control subjects

We detected a cluster of decreased gray matter volume in the left inferior frontal gyrus (IFG) in GTS patients compared to healthy controls (more details are provided in Table 2). The peak voxel of this cluster of reduced gray matter density was located in the pars triangularis of the inferior frontal cortex. The cluster itself extends into pars orbitalis.

**Table 2:** Regional gray matter volume differences in patients suffering from GTS compared to controls.

Anatomical area	L/R	Cluster size	T-value	x	y	z
**Tourette GM volume decrease compared to healthy controls**						

IFG (BA 47/12)	L	144	3.44	36	38	3

**GM volume increase of GTS+ADHD and GTS+ADHD+OCD compared to GTS+OCD**						

Amygdala	L	181	4.65	-33	-2	-20

Listed are all regions that survived a cluster size extent threshold of k > 99 voxels. Coordinates refer to the point of maximal change in MNI space

In order to be sure that the IQ score differences between groups have not confounded our results, we have conducted a separate analysis including IQ scores as covariates. We still found a cluster of reduced gray matter volume density in the left IFG. Thus, we are sure that the significant, but small IQ group differences cannot explain differences of gray matter volumes between groups.

**Figure 1:**
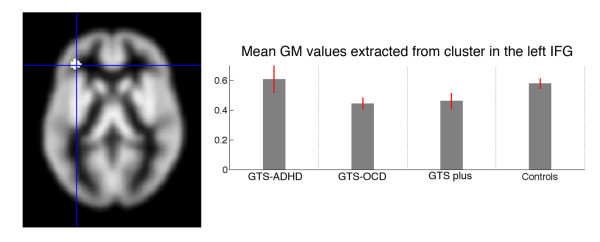
**Decreases of gray matter volumes in GTS patients**. Overlay of gray matter decreases on the SPM8 gray matter template illustrating where group comparisons revealed significantly reduced gray matter volume differences in GTS groups compared to healthy controls. Additionally, extracted gray matter values of inferior frontal gyrus for specific patient subgroups and controls are shown. The display is presented in neurological convention (R = R; L = L)

As can be seen in Figure 1, the differentiation of parameter estimates of the different GTS subgroups and normal controls revealed that the decrease in gray matter in the left IFG was mainly evoked by those two GTS subgroups suffering from comorbid OCD (GTS + OCD and GTS "plus"). IFG gray matter values of GTS patients with comorbid ADHD (but without OCD) seem to be comparable to healthy controls. However, on the background of the very small size of four patients (GTS + ADHD) and in order to test this assumption, we excluded this group from the following analysis. For the comparison of the remaining two subgroups which had comorbid OCD symptomatology (GTS + OCD and GTS "plus") with healthy controls, we found a more pronounced cluster in the left IFG (-34, 42, 7; cluster size 464 voxels, t-value 4.64). There were no regions demonstrating volume increases in GTS patients for this comparison.

### Differences in GM volumes between GTS patient subgroups

In order to show that GTS subgroups with ADHD comorbidity (n = 8 + 4) are significantly different compared to the GTS group without ADHD comorbidity (GTS and OCD; n = 17) in regard to gray matter volumes, we directly compared these groups. By comparing the subgroups with comorbid ADHD (GTS + ADHD and GTS "plus") with patients suffering from Tourette with OCD symptomatology only (GTS + OCD), we found an increase of gray matter density in the left amygdala. This cluster was also significant in comparison to healthy controls, indicating differences due to pathology. We found no significant clusters of volume reductions here.

### GM volumes: correlations with clinical scores

**Figure 2:**
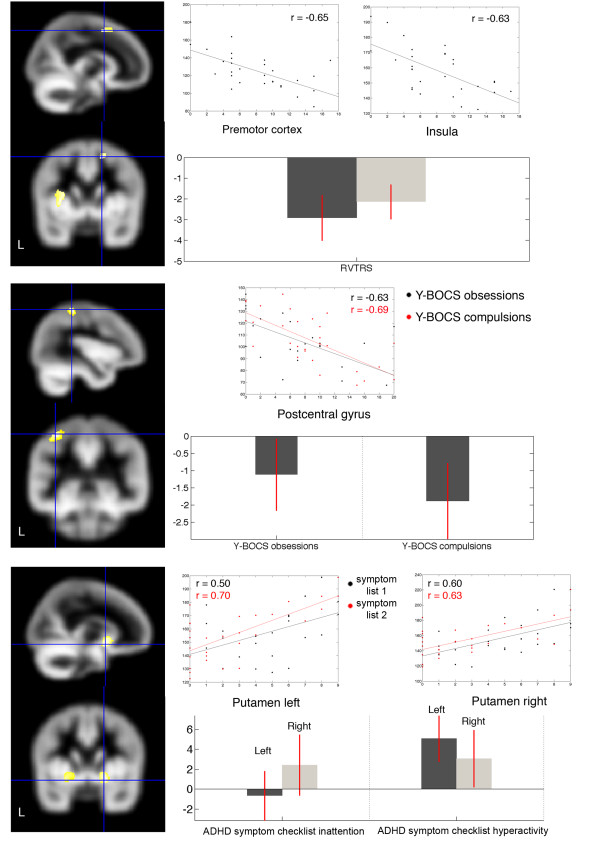
**Selected cortical regions showing significant correlations with test scores in GTS patients**. The left side shows cortical regions that were significantly correlated with the RVTRS (upper), Y-BOCS (middle), and ADHD (lower) scores (symptom checklist inattention (1) and hyperactivity (2)). The crosshair was laid on the most significant voxel. Additionally, scatterplots displaying the correlation between the clinical score (x-axis) against the globally scaled gray matter values (y-axis), and parameter estimates plotted as bar graphs are provided. All correlations were significant at our initial *p *< 0.001

Correlations between clinical scores and cortical gray matter volumes in patients suffering from GTS are presented in Figure 2 and Table 3. By separately conducting multiple regression analyses for different symptoms (tics (YGTSS and RVTRS), OCD (Y-BOCS), and ADHD (symptom checklist, CAARS, WURS-K)), we successively excluded clinical scores that decreased the fit of our model. All following results are significant at our initial *p *< 0.001, and represent multiple regression models best predicting GM values of GTS patients.

**Table 3:** Brain regions showing significant correlations with clinical scores

Anatomical area	L/R	Cluster size	T-value	x	y	z
**GM correlations with RVTRS**						

Premotor cortex	R	157	4.58	16	6	55

Insula	L	255	4.41	-31	6	4

Precuneus	R	185	4.07	3	-57	31

Posterior cingulate cortex	R	101	4.02	10	-40	25

IFG	R	96	3.87	30	28	-4

**GM correlations with Y-BOCS (total score)**						

Postcentral gyrus	L	374	5.75	-38	-39	60

Superior parietal lobule	R	184	4.96	15	-67	52

Occipital pole	R	449	4.34	22	-100	9

**GM correlations with ADHD scores (Symptom checklist, CAARS, WURS-K)**						

Putamen	L	433	4.73	-26	9	-9

	R	503	4.94	20	6	-12

**GM correlations with BDI**						

Anterior cingulate cortex	L	202	4.44	2	14	18

Middle temporal gyrus	L	90	4.34	-51	-49	-2

Lateral occipitotemporal gyrus	R	90	4.30	39	-48	-17

**GM correlations with STAI**						

Anterior cingulate cortex	L	1423	5.07	-16	47	7

Insula	R	383	5.01	48	-3	1

	L	558	4.91	-42	9	-3

Middle occipital gyrus	L	135	4.60	-39	-61	24

Lateral occipitotemporal gyrus	R	105	4.47	36	-52	-8

Midcingulate cortex	L	149	4.35	-3	-27	40

Middle frontal gyrus	R	158	4.19	26	58	1

	L	141	4.14	-30	60	1

Superior parietal lobule	R	278	4.12	0	-64	46

Medial frontal gyrus	L	132	3.91	-6	35	42

Tic severity measured with the RVTRS correlated negatively with the volume of the right superior frontal gyrus (r = -0.65) and the left insula (r = -0.63). No regions showed a positive correlation with the RVTRS.

The left postcentral gyrus showed a negative correlation with the Y-BOCS scores assessing obsessions (r = -0.63) and compulsions (r = -0.69) in patients with GTS. No regions showed a positive correlation with the Y-BOCS.

By conducting multiple regression analyses including ADHD scales (symptom checklist inattention and hyperactivity, CAARS, WURS-K), we found a positive correlation with gray matter volumes of the left (inattention: r = 0.5; hyperactivity: r = 0.7) and right putamen (inattention: r = 0.6; hyperactivity: r = 0.63) while no negative correlations could be observed.

## Discussion

The present study identified a common pattern of gray matter brain tissue alteration in actually unmedicated adult patients with GTS and comorbid OCD and/or ADHD characterized by left inferior frontal gyrus decreases of GM volumes. There were no significant differences between healthy controls and GTS patients concerning a GM volume increase. Based on our results, we suggest that the left IFG may constitute a common underlying neurological correlate of GTS with comorbid OCD and/or ADHD. As can be seen in Figure 1, gray matter volume reductions of the left IFG in the whole group of GTS patients are predominantly observed in the subgroups of GTS patients with comorbid OCD. This possibly reflects the failure to control behavior and may be a key feature of persistent GTS and OCD in adults. The finding of left IFG decreases of GM volumes is in line with data from recent studies [29] including our own examining patients with GTS "only" and demonstrating volume reductions predominantly in different frontal areas [24]. Thus, it is not surprisingly that in the present study volume reduction of the left IFG was pronounced in GTS patients with comorbid OCD/ADHD which suggests an additive effect. In particular, the left IFG has previously been associated as a key region for OCD [30]. Together with the inferior parietal cortex it serves as a network responsible for the active inhibition of attentional processes. Additionally, this network has been associated with voluntary shifts of attention across sensory modalities [31]. The precise location of this region is heterogeneous across studies, as it includes the most orbital part of the IFG and extends into the lateral orbitofrontal gyrus (sometimes used as synonyms). In OCD patients, functional alterations of this region have been observed, and a recent meta-analysis could associate these functional alterations to structural abnormalities [32]. However, the neuronal mechanisms are still unknown, and even the direction of the functional-structural relationship is controversial. In the above mentioned meta-analysis [32] and in a study by van den Heuvel and colleagues [33], the lateral orbitofrontal cortex was found to be reduced in OCD patients compared to healthy controls, whereas a more recent meta-analysis, in which functional and structural findings were combined, showed greater gray matter density of the lateral orbitofrontal cortex in OCD [34]. However, by using a different voxel-based meta-analytic method called signed differential mapping, Radua and Mataix-Cols [35] could not find any structural abnormalities of patients suffering from OCD in this area. They reported an association of gray matter volume increases in the basal ganglia with symptom severity in OCD.

But are there any functional changes of the left IFG in GTS? We are aware of only a single PET-study [36] reporting an abnormal positive coupling between the basal ganglia and the lateral orbitofrontal cortex. In fact, thirteen out of eighteen patients included in this study had comorbid OCD symptomatology suggesting that OCD pathology is a main factor of alterations in this frontal region. Our data corroborate the assumption that compensatory neuroplastic processes in terms of frontal cortex hypertrophy - that might help to compensate tics [29] as can be seen in GTS adolescents [22] - are absent or even reversed in adults with persistent tic disorders [37].

Our results of a comparison of the GTS subgroups with ADHD comorbidity to the GTS group without ADHD comorbidity (GTS and OCD) revealed a volume increase of the left amygdala which could also be observed in comparison to healthy controls. Structural alterations of the amygdala -gray matter volume increase in particular [12] - have been previously associated with compensatory mechanisms in GTS [38]. The findings in pure ADHD adult groups have been equivocal so far [39]. In children with GTS and comorbid ADHD symptomatology, a linkage between amygdalar volume reductions has been reported [11]. It seems reasonable to assume that our finding of gray matter volume increase in the left amygdale reflects long-term structural mechanisms in order to compensate a delay in cortical maturation during childhood [40].

VBM analyses reflect a number of anatomical features, including gray matter alterations and shifts in gyral or sulcal anatomy. Therefore, it can be speculated that GM volume decreases observed in this study might be the consequence of malformated cortical development, such as abnormal neuronal migration. Future studies using different volumetric measurements such as an estimation of cortical thickness may further contribute to the underlying structural differences in GTS [19,41].

### Lack of findings in previously reported regions and limitations of the study

We failed to detect group differences in regions previously associated with GTS pathology including the basal ganglia [7-10] and the mesencephalon [13,14]. These discrepancies may reflect sample characteristics (since we included only actually unmedicated adults GTS patients with comorbid OCD/ADHD) and different methodological approaches. For example, Ludolph [8] and Garraux [14] used predefined regions of interest and subsequently conducted small volume corrections. This analysis strategy assumes stationary smoothness, which may not even be appropriate for VBM [42]. Additionally, it has been discussed that larger sample sizes (including up to 70-90 subjects) may be a prerequisite in order to detect volume differences of small subcortical structures [43]. Thus, our failure to detect significant differences in these particular regions does not necessarily prove that these regions are indeed unaffected.

A limitation of the study was the small subgroup of only 4 patients with comorbid ADHD (without OCD). When excluding this subgroup from additional analyses, differences between GTS patients and controls even increase strengthening our assumption that GM volume reduction in the inferior frontal gyrus are mainly based on the presence of comorbid OCD. However, future structural studies including larger samples of patients with comorbid ADHD are needed to better clarify the impact of comorbid ADHD.

Although the group of patients and the control group were not comparable in regard to IQ, we do not think that group differences can be explained by these small IQ differences, since - to the best of our knowledge - there are no VBM studies available reporting morphological alterations on the basis of small group differences in intelligence. Our results are in line with recent findings in children demonstrating that IQ scores in patients with GTS "plus" (with comorbidities) are slightly reduced compared to healthy [44].

Although all patients included in this study were unmedicated for at least 6 months prior to MRI imaging, some patients were medicated with typical or atypical antipsychotics before. In patients with Tourette syndrome, it has been demonstrated that neuroleptic medication increases both caudate and globus pallidus volumes [10]. Because in patients with schizophrenia it has been demonstrated that medication effects are reversible [45,46], we speculate that in the present study possible influences of antipsychotic medication on brain volumes can be excluded. However, to the best of our knowledge, in patients with GTS no longitudinal studies are available investigating neuroleptic-induced volume changes after withdrawal from medication.

### Gray matter volume correlations with clinical scores

We found several significant correlations between GM volumes and different clinical scores. Tic severity (RVTRS) correlated negatively with GM volumes of both the left insula and the right premotor cortex. Although, video-based tic ratings represent only a short period of time, they are regarded as the most objective tic measurement. Since in adult patients, spontaneous tic fluctuations are less marked compared to children, it can be speculated that the RVTRS indeed represents tic severity in an individual patient.

This result is in line with recent morphological [47] and functional [48,49] findings in GTS patients emphasizing an involvement of the insula in tic generation. Furthermore, in people with persistent and routine habits an involvement of this brain area has been found supporting the hypothesis of an insula-striatal neural interplay during the preference of default behavior [50]. The premotor cortex has also been described before as a brain region that is involved in GTS pathology demonstrating abnormal metabolic networks [51]. Additionally, it was recently suggested that cortical thinning in premotor areas is correlated with more complex tics in GTS adults [19].

In line with findings of a recent study in patients with "pure" OCD [52], we found a negative correlation between the severity of OCD (Y-BOCS) and the left postcentral gyrus. In "pure" OCD an involvement of different brain areas including both the (orbito-) frontal cortex and the anterior cingulate cortex, as well as subcortical structures such as the thalamus and caudate nucleus has been suggested [53,54]. However, there is evidence that in different symptom dimensions of OCD different brain networks might be involved [55]. For example, functionally decreased activity in the left postcentral gyrus has been found in patients with obsessions/checking rituals but not in those with cleanliness/washing rituals [53]. It is well known that in patients with GTS plus OCD a different OCD symptom subtype occurs compared to patients with "pure" (tic-free) OCD: While obsessions/checking rituals are relatively common in GTS patients, aggressive repetitive thoughts, contamination worries and washing behaviours are rare [56]. Thus, our finding of a negative correlation between OCD severity and GM volume of the left postcentral gyrus further supports an involvement of this brain area in the pathology of OCD in GTS patients. In addition, it can be speculated that comparable brain areas are involved in those patients with the OCD subtype obsessions/checking rituals and GTS patients with comorbid OCD suggesting a common underlying pathophysiology.

In contrast, we found a positive correlation between the severity of ADHD (assessed by the symptom checklist, CAARS, and WURS-K) and bilateral GM volumes in the putamen. This finding is in line with results obtained from recent MRI studies in children with "pure" ADHD consistently demonstrating an involvement of the putamen [57,58] with unilateral or bilateral GM volume reduction in this brain area. Structural studies involving adults with ADHD are limited and resulted in contradictory data (for an overview see [59]). It can be speculated that differences regarding GM volume sizes in children with ADHD compared to adults with ADHD are related to developmental neuroplastic processes. Comparable findings with opposite volume changes in children and adults have been demonstrated in patients with GTS [24,29]. Our results further support the hypothesis that the basal ganglia, and in particular the putamen, are involved in the pathophysiology of ADHD, since this region has been associated with diverse cognitive functions (e.g., language, learning and memory, attention and control of behavioural responses) [59]. In addition, our finding of a correlation between ADHD severity and GM volume of the putamen in patients suffering from GTS is in line with the assumption that the coexistence of ADHD and GTS represents an additive rather than an interactive or phenotype model [60].

## Conclusions

By means of VBM we found a gray matter reduction in the left IFG in GTS patients with comorbid OCD/ADHD compared to healthy controls. Furthermore, the volume of the left amygdala was found to be increased in the subgroup of GTS patients with ADHD symptomatology. From our data it is suggested that the coexistence of tics and ADHD and/or OCD represents an additive model, since in patients with GTS "plus" severity of tics, ADHD, and OCD each correlated with those brain regions (left insula and the right superior frontal cortex, putamen, left postcentral gyrus, respectively) that are also involved in "pure" GTS, "pure" ADHD and "pure" OCD.

## Methods

### Participants

Forty-one adult (≥ 18 years) male patients with the diagnosis of GTS according to DSM IV-TR criteria were scanned for this study. All patients suffered either from comorbid OCD, ADHD, or both. All patients were actually unmedicated for at least six months before entering the study. For the diagnosis of ADHD we used both, a psychiatric examination and different self-rating scales including the DSM-IV symptom check-list for ADHD [61] consisting of 18 screening questions for inattention and hyperactivity (9 questions each), the Conners' Adult ADHD Rating Scales (CAARS; Self report - long version) [62], consisting of 66 items assessing for inattention/memory problems, impulsivity/emotional lability, hyperactivity/restlessness, and problems with self-concept, and the Wender-Utah-Rating-Scale short form (WURS-K) [63], a 25-item-self-rating for the retrospective diagnosis of ADHD in adults. To diagnose and rate OCD, we used a psychiatric evaluation as well as the German versions of the Yale-Brown Obsessive Compulsive Scale (Y-BOCS) [64], a measurement for OCD containing a checklist for specific obsessions and compulsions as well as a rating scale separately for obsessions and compulsions (scoring for expenditure of time, interference, distress, resistance, degree of control). Tic severity was rated using the Yale Global Tic Severity Scale (YGTSS) [65], consisting of ratings separately for motor and vocal tics concerning number, frequency, intensity, complexity, and interference, plus an overall TS impairment rating as well as the modified Rush Video-based Tic Rating Scale (RVTRS) [66]. The RVTRS is based on a 10-minute film protocol including near and far body views rating on five disability categories (number of body areas, frequency of motor tics, frequency of vocal tics, severity of motor tics, and severity of vocal tics). In addition, IQ was assessed with the MWT-B (Mehrfachwahl-Wortschatz-Intelligenztest), a test in which a real word among four pseudo-words has to be identified [67]. In order to assess depression and anxiety, Beck Depression Inventory-II (BDI), consisting of 21 questions about the subject's feeling in the last week) [68], and STAI (State-Trait Anxiety Inventory), consisting of 40 questions for measuring both "state anxiety" and "trait anxiety" in adults [69] were used.

None of the patients had a history of head trauma, epilepsy, brain surgery, systemic illness, drug or alcohol abuse, or any other significant comorbid disorder. In all patients a neurological and psychiatric examination was performed by one of the authors (KR MV) who is experienced in the diagnosis of TS, OCD, and ADHD. Twenty-five healthy control subjects individually matched for age and gender were enrolled in this study. Exclusion criteria were the same as for the patients' group. Healthy controls were interviewed and examined in the same way as patients. Handedness for all participants was assessed with the Edinburgh Handedness Inventory [70]. The study was approved by the local ethical standards committee and was carried out in accordance with the declaration of Helsinki. All participants gave written informed consent after all procedures had been fully explained to them before entering the study.

Of 66 participants included in the study, data sets of 53 (n = 29 GTS patients, n = 24 healthy controls) participants were used for further analyses. Data sets of 13 participants did not survive our strict image quality control due to different reason: poor image quality due to excessive movement artifacts (n = 9 GTS patients), anatomical abnormalities that prevent reliable and accurate spatial normalization (n = 1 healthy control), and claustrophobia leading to incomplete measurements (n = 3 GTS patients). Demographic and clinical parameters are summarized in Table 1. For further analyses patients were divided into three different subgroups according to their kind and number of comorbid disorders: (1) GTS "plus" (with comorbid OCD *and *ADHD, n = 8), (2) GTS-OCD (n = 17), and (3) GTS-ADHD (n = 4).

### MR image acquisition

All MRI scans were obtained at 1.5 Tesla General Electric Signa Horizon LX (General Electric Company, Milwaukee, WI, USA). A high-resolution three-dimensional T1-weighted spoiled gradient recalled echo (SPGR) sequence generated 124 contiguous sagittal slices (TR = 24 ms; TE = 8 ms; flip angle = 30°; voxel dimensions 0.97 × 0.97 × 1.5 mm^3^).

### Voxel-based GM volume analysis

Data were processed and examined using the SPM8 software (Wellcome Department of Imaging Neuroscience Group, London, UK; http://www.fil.ion.ac.uk/spm, where we applied VBM standard routines and default parameters implemented in the VBM8 toolbox (r347) http://dbm.neuro.uni-jena.de/vbm.html running under the MATLAB 7.10 (R2010a; Mathworks, Sherbon, Massachusetts) environment. Voxel-based morphometry is a whole-brain, unbiased, semi-automated technique for characterizing regional cerebral differences in structural magnetic resonance images [28]. Before segmentation into gray and white matter segments, the anterior commissure was manually defined. Images were then normalized, bias field corrected, and tissue classified. Subsequently, analyses were performed on GM segments resulted in the DARTEL (Diffeomorphic Anatomical Registration Through Exponentiated Lie Algebra) analysis, which were multiplied by the non-linear components derived from the normalization matrix in order to preserve actual GM values locally. This procedure allows for comparing the absolute amount of tissue corrected for individual brain sizes. The rationale behind DARTEL is to increase the accuracy of inter-subject alignment by modeling the shape of each brain using millions of parameters (three parameters for each voxel). This procedure has been shown to improve the parameterization of brain shapes [71]. Images were smoothed with an 8 mm full-width at half-maximum (FWHM) isotropic Gaussian kernel to make the data more normally distributed and to compensate for the inexact nature of spatial normalization. By applying independent t-tests we examined differences of demographic data and clinical variables between patients suffering from GTS and healthy controls. An ANOVA was conducted to calculate voxel-wise GM differences. To avoid possible edge effects between different tissue types, we excluded all voxels with values of less than 0.1 (absolute threshold masking). Correlations of gray matter parameter estimates of patients with clinical scores were calculated using multiple regression models in SPM8. Statistical outcomes were corrected for multiple comparisons using an empirically determined extent threshold at an uncorrected *p *< 0.001. This method is implemented in SPM8 and refers to the estimated smoothness of the images. After determining of the number of resels, the expected Euler characteristic is calculated. This is used to give the correct threshold (number of voxels) that is required to control for false positive results.

Resulting parameter estimates were extracted from significant clusters and scaled to the global mean with the REX toolbox http://web.mit.edu/swg/software.htm.

## Authors' contributions

MW performed the analyses and drafted the manuscript. SB participated in the design of the study and the collection of data. TP and JG participated in data collection, and reviewed the manuscript. AG, NB, HB, and RD reviewed the manuscript. KRM-V participated in study design and coordination and edited the manuscript. All authors read and approved the final manuscript.
